# SIMULTANEOUS LAPAROSCOPIC INGUINAL HERNIA REPAIR AND CHOLECYSTECTOMY: DOES IT CAUSE MESH INFECTION?

**DOI:** 10.1590/0102-672020210002e1600

**Published:** 2021-10-18

**Authors:** Christiano Marlo Paggi CLAUS, João Rafael Bora RUGGERI, Eduardo Brommelstroet RAMOS, Marco Aurelio Raeder COSTA, Leonardo ANDRIGUETTO, Alexandre Coutinho Teixeira de FREITAS, Júlio Cezar Uili COELHO

**Affiliations:** 1 Minimally Invasive Surgery/Jacques Perissat Institute, Positivo University, Curitiba, PR, Brazil; 2 Department of Surgery, Hospital Nossa Senhora das Graças, Curitiba, PR, Brazil; 3 Department of Surgery, Federal University of Paraná, Curitiba, PR, Brazil

**Keywords:** Hernia, inguinal, Cholecystectomy, Laparoscopy, Hérnia inguinal, Colecistectomia, Laparoscopia

## Abstract

**Background:**

Repair of inguinal hernia concomitant with cholecystectomy was rarely performed until more recently when laparoscopic herniorrhaphy gained more adepts. Although it is generally an attractive option for patients, simultaneous performance of both procedures has been questioned by the potential risk of complications related to mesh, mainly infection.

**Aim:**

To evaluate a series of patients who underwent simultaneous laparoscopic inguinal hernia repair and cholecystectomy, with emphasis on the risk of complications related to the mesh, especially infection.

**Methods:**

Fifty patients underwent simultaneous inguinal repair and cholecystectomy, both by laparoscopy, of which 46 met the inclusion criteria of this study.

**Results:**

In all, hernia repair was the first procedure performed. Forty-five (97,9%) were discharged within 24 h after surgery. Total mean cost of the two procedures performed separately ($2,562.45) was 43% higher than the mean cost of both operations done simultaneously ($1,785.11). Up to 30-day postoperative follow-up, seven (15.2%) presented minor complications. No patient required hospital re-admission, percutaneous drainage, antibiotic therapy or presented any other signs of mesh infection after three months. In long-term follow-up, mean of 47,1 months, 38 patients (82,6%) were revaluated. Three (7,8%) reported complications: hernia recurrence; chronic discomfort; reoperation due a non-reabsorbed seroma, one in each. However, none showed any mesh-related complication. Satisfaction questionnaire revealed that 36 (94,7%) were satisfied with the results of surgery. All of them stated that they would opt for simultaneous surgery again if necessary.

**Conclusion:**

Combined laparoscopic inguinal hernia repair and cholecystectomy is a safe procedure, with no increase in mesh infection. In addition, it has important advantage of reducing hospital costs and increase patient’ satisfaction.

## RESUMO

**Racional:**

Reparo da hérnia inguinal concomitante à colecistectomia era raramente realizado até mais recentemente, quando a herniorrafia laparoscópica ganhou mais adeptos. Embora geralmente seja opção atraente para pacientes, a realização simultânea tem sido questionada pelo risco potencial de complicações relacionadas à tela, principalmente infecção.

**Objetivo:**

Avaliar uma série de pacientes submetidos a colecistectomia e herniorrafia inguinal laparoscópica simultâneas, com ênfase no risco de complicações relacionadas a tela, em especial infecção.

**Métodos:**

Cinquenta pacientes foram submetidos a herniorrafia e colecistectomia simultâneas por videolaparoscopia, dos quais 46 atenderam aos critérios de inclusão.

**Resultados:**

Em todos, a herniorrafia foi realizada inicialmente. Quarenta e cinco (97,9%) tiveram alta em 24 h. O custo médio total dos dois procedimentos realizados separadamente ($2.562,45) foi 43% maior do que o custo médio das duas operações feitas simultaneamente ($1.785,11). Após 30 dias de acompanhamento pós-operatório, sete (15,2%) apresentaram complicações menores. Após três meses, nenhum necessitou de readmissão hospitalar, drenagem, antibioticoterapia ou sinal de infecção de tela. No seguimento em longo prazo, média de 47,1 meses, 38 (82,6%) foram reavaliados. Três (7,8%) relataram complicações: recorrência de hérnia; desconforto crônico; reoperação por seroma não reabsorvido. No entanto, nenhum apresentou qualquer complicação relacionada à tela. Questionário de satisfação revelou que 36 (94,7%) ficaram satisfeitos com o resultado da operação. Todos afirmaram que optariam pela ela simultânea novamente se necessário.

**Conclusão:**

O reparo da hérnia inguinal concomitante com colecistectomia por laparoscopia é procedimento seguro, sem aumento de infecção da tela. Além disso, tem a vantagem importante de reduzir custos hospitalares e aumentar a satisfação do paciente.

## INTRODUCTION

Inguinal hernia repair and cholecystectomy are two of the most common surgical procedures performed worldwide^17^ ^,^ ^21^. During the open surgery era, they were rarely performed simultaneously, since these procedures were performed through distinct accesses.

However, with large-scale adoption of the laparoscopic technique by general surgeons, possibility of treating both diseases in a single surgical procedure had been considered^2^ ^,^ ^8^ ^,^ ^16^ ^,^ ^20^. The concept appeals to patients who see benefits in having a single anesthesia and recovery time.

Nevertheless, many surgeons have questioned this approach. The first point of criticism comes from dealing with two different surgical fields (upper abdomen and inguinal region) with different surgical positions and ports placement. Second, some authors have advocated against combined surgery due to greater potential risk of mesh infection. Laparoscopic inguinal hernia repair (LIHR) with a mesh is a clean procedure while laparoscopic cholecystectomy (LC) is potentially contaminated, since patients with cholelithiasis present higher prevalence of bile contamination by *Escherichia coli*, *Klebsiella pneumoniae* and other microorganisms^12^ ^,^ ^13^ ^,^ ^23^ ^,^ ^27^.

Despite these doubts, there are only few publications that addressed the question: Is it safe to perform LIHR and LC simultaneously in terms of mesh complications? 

The aim of this study was to evaluate a series of patients who underwent simultaneous LIHR + LC, with emphasis on the risk of complications related to the mesh, especially infection.

## METHODS

Ethical Committee approved study and all patients signed an informed consent to have access to their clinical file and participate in the study (Number 0001).

From January 2010 to January 2020, a total of 50 patients underwent concomitant LIHR+LC at the General Surgery Division of Hospital Nossa Senhora das Graças, Curitiba, PR, Brazil. Indication for surgery was established based on the simultaneous diagnosis of inguinal hernia and symptomatic cholelithiasis. Four patients were excluded from the study due to intraperitoneal mesh placement (n=1), gallbladder polyp (n=1) and absence of minimum postoperative follow-up of three months (n=2). The remaining 46 were enrolled.

### Surgical technique

All patients underwent primarily the inguinal repair, TEP (totally extraperitoneal) or TAPP (transabdominal preperitoneal) according to surgeons’ preference followed by laparoscopic cholecystectomy. Patient and surgical team position and trocars placement were performed according to standard technique previously described by our group^4^ ^,^ ^6^.

For cholecystectomy, two trocars (subxiphoid and subcostal) were added while the umbilical and right flank trocars were just redirected to the upper abdomen. For inguinal repairs, a heavyweight mesh (14/15 cm x 11/12 cm) was used to cover the myopectineal orifice. Mesh was fixed with absorbable tacks in all TAPPs and only on large direct hernias (M3 according to European Hernia Society classification) in TEPs^14^. In TAPP, a tight closure of the peritoneum was performed with continuous absorbable suture to avoid possible contamination of the mesh due to bile leakage. In TEP, all possible holes in the peritoneum were closed with suture or clips. For LC, after establishing the critical view of safety, double proximal clipping of cystic duct and cystic artery were performed followed by dissection of the gallbladder bed with monopolar energy. Gallbladder was removed through the umbilical trocar (protected by bag in the minority of cases, according to the option of the surgeons), which was closed with absorbable suture.

### Perioperative evaluation

Intraoperative complications such as bleeding, bile leakage or conversion to open procedure were evaluated. The gallbladder macro and microscopic aspect was considered. Operative time, length of hospital stay, hospital costs (in January 2020, the reference value used was US$ 1 equal to R$ 4) and readmission were also assessed. Patients were evaluated after 7, 30 and 90 days postoperatively and in sequence as needed. Medical records were retrospectively analyzed.

### Follow-Up

In March and April of 2020, patients were revaluated at the doctor’s office or contacted by phone. Any related surgical complication (re-admission, recurrence, new surgical procedure) especially regarding to mesh infection (antibiotic therapy) was assessed. Hernia recurrence inventory for inguinal hernia was applied to patients^29^. Those with at least one positive response were evaluated by a surgeon. In a simplified surgical satisfaction questionnaire, patients were asked to answer whether they were satisfied, neutral or dissatisfied with the outcome of the surgery and if they would opt or not for the combined surgery again.

## RESULTS

Mean age of the 46 patients was 63.5 years (35-87) and 40 (86,9%) were male. TEP was the most employed technique for inguinal repair (n=42, 91.3%) while TAPP was performed in only four (8.7%). A total of 63 inguinal hernias were repaired (29 were unilateral and 17 bilateral). Patient’s characteristics, including findings regarding the hernias and gallbladder are shown in Table 1.

**TABLE 1:** Clinical characteristics of patients

	n	%
ASA score 1 2 3	16 27 3	34.8 58.7 6.5
Hernia Unilateral Bilateral Total	29 17 63	
Hernia classification Indirect Direct Combined Femoral	30 26 6 1	47.6 41.3 9.5 1.6
Hernia recurrence (open anterior repair)	6	9.5
Gallbladder Chronic cholecystitis Acute cholecystitis Escleroatrofic cholecystitis	44 1 1	95.6 2.2 2.2

Mean operative time was 111 min (60-210). There were no major complications during surgery as well as no conversion to the open technique. During hernia repair, difficulty in creating space in TEP occurred in two patients and one of them needed conversion to TAPP. No other complications have been observed. Holes in the peritoneum or minimum bile leakage confined to the subhepatic area that was rapidly aspirated were not considered intraoperative complications. Forty-five patients (97,9%) were discharged within 24 h after surgery (19 at the same day). Only one was hospitalized for longer period (six days) requiring blood transfusion due to previous coagulopathy secondary to hematological disease.

Cost of surgery (LIHR+LC) was analyzed and compared with cost of 46 who underwent LIHR and 46 LC separately, in the same period of the study, who were selected at random. Mean cost of LC and LIHR separately was $1,370.41 (1,028.88-1,985.52) and $1,192.04 (950.61-1852.21) respectively. When the cost of the two procedures were added, the mean cost was $ 2,562.45 (1,979.49-3,837.73) and it was 43% higher than the mean cost of both operations performed simultaneously, $1,785.11 (1,454.28-2,597.30).

Up to the 30-day postoperative follow-up, seven patients (15.2%) presented complications: three sero-hematomas; two testicular edema/ischemic orchitis; one superficial wound infection (umbilical trocar) and one urinary retention. Up to 3-month follow-up, two (4,3%) remained with local volume in the inguinal region compatible with seroma and one (2.17%) complained of persistent/chronic pain. No patient required re-admission, percutaneous drainage and antibiotic therapy or presented any other signs of mesh infection.

With mean follow-up of 47,1 months (3-121), 38 (82,6%) were revaluated (five lost follow up and three died due to other medical conditions). In long-term follow-up, three (7,8%) reported complications: one had hernia recurrence (2,6%), one chronic discomfort without activity limitation and one needed to be reoperated for a non-reabsorbed seroma (pseudo-hydrocele). However, no patient showed any mesh-related complications signs, including infection According to the satisfaction questionnaire, 36 patients (94,7%) were satisfied with the results of the surgery. All of them stated that they would opt for simultaneous surgery again if necessary.

## DISCUSSION

Cholecystectomy and inguinal hernia repair are two of the most common surgical procedures performed by general surgeons worldwide. Laparoscopic approach for cholecystectomy, described in the mid-1980s, quickly gained enthusiasts and, in the early 1990s, was considered the standard approach to perform cholecystectomy^11^ ^,^ ^26^. The same did not happen with inguinal hernia repairs. Despite being described in the early 90’s, both TAPP and TEP took longer time to be accepted by most surgeons^2^ ^,^ ^15^. Only in recent years, after several studies demonstrated significant advantages of laparoscopic access, this technique has been widely employed^5^ ^,^ ^7^ ^,^ ^9^.

As both diseases are highly prevalent, they may occur concomitantly. The fact of needing two different operative fields and, for a long time, two different approaches (open for inguinal hernia repairs and laparoscopic for cholecystectomy), these two procedures were rarely performed simultaneously. However, with the greater acceptance of minimally invasive inguinal hernia repair, this possibility has now been employed.

The key points in favor of combined surgery are related to patients’ perspectives. Most patients prefer to solve both problems at the same time, with a single anesthesia and only a period of recovery. Thus, physical and professional activities are restricted for only one period. Of the 38 patients who responded our questionnaire, all of them stated that they would opt for simultaneous surgery again. In general, the risk of long-term complications for combined surgery does not appear to be greater when compared to each surgery separately. In our series, 7,8% of the patients presented long-term complications: one recurrence, one chronic discomfort without limitation and one seroma that needed reintervention. These results are no different from those published in the literature after LIHR isolated^1^ ^,^ ^7^ ^,^ ^9^. Overall satisfaction rate with surgery was 94,7%.

Another benefit comes from the economic standpoint since the costs of a single hospitalization are generally lower than two-stage procedures. Hayakawa et al reported lower costs for combined LIHR+LC when comparing to the sum of the two procedures separated^8^. Hospital stay for patients undergoing LIHR+LC is similar to that for the ones undergoing separate LC and LIHR. In our institution, the mean length of hospital stay was 1,1 days (97,9% were discharged within 24 h). We compared the cost of simultaneous LIHR+LC with costs of either LC or LIHR performed separately. Mean costs of LIHR+LC was $1.785,11 compared with $1.370,41 for LC and $1.192,04 for LIHR. This indicates that combined procedure can provide an important economic benefit. In our study, combined procedure was on average 30% cheaper than that of the sum of the two procedures separately. Additionally, simultaneous LIHR+LC seems to have similar costs when compared to LC plus open inguinal hernia repair, since general anesthesia and laparoscopic equipment will both be used anyway^22^.

Meanwhile, arguments against simultaneous surgery are: two distinct operative fields - upper abdomen and inguinal region, different patient and surgical team position and different trocars placement. In addition, an important point that has been raised against simultaneous surgery is the potential greater risk of mesh contamination. Large series have been published showing that mesh infection rates after LIHR are less than 1%, and in many studies even zero^25^ ^,^ ^28^. LIHR is a clean surgery with prosthesis while LC is a potentially contaminated surgery. Although bile is considered sterile in normal individuals, in patients with cholelithiasis the presence of bacteria in bile occurs in up to 50% of the cases^12^ ^,^ ^27^. Most commonly found pathogens are *Escherichia coli*, *Klebsiella sp.* and *Pseudomonas sp.* ^23^. In our study, all patients underwent elective surgery, and in only one, signs suggestive of acute cholecystitis were found during the procedure.

This concern has been demonstrated by many surgeons worldwide in various social media discussion groups such as International Hernia Collaboration. However, there is little evidence in the literature to support this theory. Previous publications by Hayakawa^8^ and Quezada^20^ did not report any case of mesh infection in patients undergoing combined TAPP + LC, with a median follow-up of 40 months. In our series, the largest published so far to the best our knowledge, no cases of mesh-related complications were seen in a mean follow-up of 47 months. Sarli et al^22^ in a randomized study did not report any difference in terms of postoperative complications, recurrence or mesh infection when comparing open vs. laparoscopic inguinal hernia repair followed by LC. Likewise, there seems to be no advantage in performing an open inguinal hernia repair simultaneously to LC.

Extrapolation of this concept of inguinal repair combined with other procedures should be done with caution. Praveen Raj et al^18^ reported an incidence of mesh infection of 0.8% when cholecystectomy or hysterectomy was associated with intraperitoneal onlay mesh repair (IPOM). Meanwhile, mesh infection has been reported up to 5% after bariatric surgery (sleeve/vertical gastrectomy) combined with ventral hernia repair with IPOM mesh^3^ ^,^ ^19^.

The type of inguinal hernia repair technique can be discussed in this scenario of combined surgeries. The fact that access to peritoneal cavity will be necessary to perform LC, TAPP, which is the most commonly used technique in hernia repair worldwide, could be the preferred technique to be used. On the other hand, TEP has an important advantage: it maintains the integrity of the peritoneum because there is no opening of peritoneum in the majority of cases. Presumably, this could be a better protection of the mesh. However, no difference in the risk of mesh infection between the two techniques has been reported. Savita et al^24^ in a series of 23 patients reported no difference between TEP (n=11) and TAPP (n=12) after LC regarding the risk of infectious complications. When TAPP technique is used, special attention should be given to proper closure of the peritoneum. In our service, TEP is the preferred technique even in isolated inguinal hernia repair.

We performed inguinal repair before cholecystectomy. The fact that LIHR is performed before than LC prevents contamination of the mesh by surgical instruments. In our series and most published articles, except for the Chilean group, this is the usual sequence^8^ ^,^ ^10^ ^,^ ^20^. In cases where it is chosen to perform the LC first, significant bile leakage into the cavity should urge the surgeon not to proceed with LIHR and leave it for a future procedure. An alternative is to perform open repair, Lichtenstein type, or even repair without a mesh. However, these issues should be properly discussed with patients in the preoperative period.

The major limitation of our study is the retrospective evaluation. This is minimized because all surgical procedures were performed by the same surgical team and the data were retrieved from electronic medical records and study protocols. Although the number of patients evaluated is limited, our series is the largest in the literature.

## CONCLUSION

Combined laparoscopic inguinal hernia repair and cholecystectomy is a safe procedure, with no increase in the rate of mesh-related complication, especially infection. In addition, it has the important advantage of reducing hospital costs and increase patient’ satisfaction.
